# Analysis of the Ni-5%at.W Alloy Substrate Texture Evolution at Different Strain Levels Using the EBSD Technique

**DOI:** 10.3390/ma17215334

**Published:** 2024-10-31

**Authors:** Xufeng Wang, Hongli Suo, Yaotang Ji, Zili Zhang, Lanjin Wang, Lei Wang, Jianhua Liu, Qiuliang Wang

**Affiliations:** 1 Key Laboratory of Advanced Functional Materials, Ministry of Education, College of Materials Science and Engineering, Beijing University of Technology, 100 Pingleyuan, Chaoyang District, Beijing 100124, China; wangxf1233@emails.bjut.edu.cn (X.W.); 13161098797@163.com (L.W.); 2 School of Materials Science and Engineering, Liaocheng University, Liaocheng 252059, China; jiyaotang@lcu.edu.cn; 3 Institute of Electrical Engineering, Chinese Academy of Sciences, Beijing 100190, China; wanglei@mail.iee.ac.cn (L.W.); liujianhua@mail.iee.ac.cn (J.L.); qiuliang@mail.iee.ac.cn (Q.W.); 4 University of Chinese Academy of Sciences, Beijing 100049, China

**Keywords:** functional alloys, annealing, nucleation and growth, texture, electron backscatter diffraction

## Abstract

In this paper, the texture evolution of the Ni-5%W alloy baseband with different strain variables (*ε*_vM_ = 3.9, 4.9, and 5.1) during rolling and annealing was studied using the electron back scattering diffraction (EBSD) technique. The results indicate that after high-temperature annealing at 1150 °C, all three strain levels of the alloy substrates can achieve a strong cubic texture, with a content exceeding 99% (<10°). However, the texture evolution trajectory is significantly influenced by the strain level. When the content of cubic texture in the alloy substrates under strain levels of 3.9 and 5.1 is the same, significant temperature differences exist. Additionally, the different strain levels result in varying nucleation rates and growth rates of cubic texture in the Ni-5%W alloy substrates. The study reveals that in the alloy substrates under strain levels of 3.9 and 4.9, recrystallized cubic grain nuclei grow within a layered structure, resulting in larger grain sizes and lower nucleation rates. In contrast, in the alloy substrates under a strain level of 5.1, recrystallized cubic grain nuclei form from small equiaxed grains, leading to higher nucleation rates but smaller grain sizes, competing with random orientations. In the later stages of nucleation, recrystallized grains in the alloy substrates under a strain level of 5.1 exhibit a significant size advantage, rapidly growing by engulfing randomly oriented grains. Compared to the alloy substrates with lower strain levels, the recrystallized cubic grains in the alloy substrates under a strain level of 5.1 have higher nucleation rates and faster growth rates.

## 1. Introduction

The high-temperature superconductor REB2aCu3O7-x (REBCO, RE = rare earth) offers advantages such as a high critical current, high critical transition temperature, and high critical magnet. REBCO is widely used in various applications, including cables, fault current limiters, generators, maglev, and fusion magnets [1,2,3,4]. However, due to the poor flexibility of the superconducting layer, typically a ceramic material, it is necessary to epitaxially grow this layer on a flexible metal substrate, resulting in a “sandwich” multilayer composite structure. The metal substrate not only provides mechanical support for the superconducting layer but also facilitates the layer-by-layer conduction of texture to the superconducting layer [5]. Nickel alloys, due to their low cost, are commonly chosen as substrates for preparing coated conductors, facilitating the conduction of the cube texture through the RABiTS route [6,7,8]. Zhang Y.H. et al. [9] observed that annealing pure nickel above 600 °C leads to abnormal grain growth and instability in the cube texture. This issue can be mitigated through alloying, and the texture improves with increasing annealing temperature. In NiW alloys, the stacking fault energy of the alloy gradually decreases with increasing W content. This energy level significantly impacts the cooling organization of NiW alloys, resulting in a reduced cube texture after recrystallization annealing. As a result, the Ni-5%W alloy substrate currently stands as the predominant alloy substrate for coated conductor REBCO [10,11,12]. However, existing research on Ni-5%W substrates has primarily concentrated on enhancing the annealing process to achieve a robust cube texture. Notably, there is a lack of comprehensive investigation into the nucleation mechanism during the annealing process and the evolution of the texture of Ni-5%W alloy substrate rolling texture and annealing process under varying large strains. Furthermore, there is a need to explore the temperature decay caused by the texture reaching the same level under different strain conditions.

The alloy undergoes intense deformation to form a rolling texture. Metals with rolling texture develop a recrystallized texture after recrystallization annealing [13,14,15,16]. Both the deformation [17,18,19,20,21] and annealing processes [22,23] play a crucial role in texture evolution. In the 1840s, Barrett [24] and Beck [25] proposed the theories of oriented nucleation and oriented growth for the formation of recrystallized cube texture. These theories have been widely applied to describe the formation of recrystallized texture in many metals. In 2009, Professor Penelle Richard [26] of the University of Paris XI, France, noted that the nucleation mechanism of recrystallized cube-oriented grains in face-centered cubic (FCC) metals remains unclear. TEM studies did not detect a 40° <111> relationship between the cube-oriented grains and the non-cubic revertant tissues, and there is no way to demonstrate the orientation growth mechanism of the cube-oriented grains.

In recent years, researchers have conducted numerous studies on the effects of cold rolling and annealing on FCC alloys. Shi-Hoon Choi et al. [27] at Soon Chun National University, Korea, observed microstructural inhomogeneity along the ND direction in initially hot-rolled AA5083 plates, which persisted in both primary cold-rolled and intermediate annealed specimens. However, no inhomogeneity along the ND direction was observed in the secondary cold-rolled and final annealed specimens. Shabani A. et al. [28] found that FeCrCuMnNi multiphase high-entropy alloys under different annealing regimes did not undergo phase transformation, but the FCC 1 phase recrystallized earlier compared to the FCC 2 phase and the FCC 2 phase started to recrystallize mainly due to nucleation and inhomogeneity at grain boundaries. Baoqi Guo et al. [29] from Kyoto University, Japan, investigated the formation of static recrystallization texture during the annealing of CoCrFeMnNi high-entropy alloys with different cold-rolled deformations. They analyzed the microstructure evolution during the annealing process using the quasi-in situ electron backscattering diffraction (EBSD) technique and observed that the texture persisted after the initial recrystallization but appeared to be weakened. Furthermore, they identified the shear zone in the texture as the preferred nucleation site for recrystallization.

As mentioned above, researchers have conducted fundamental studies on the formation of a strong cube texture during the deformation rolling and annealing of alloys. However, a systematic study comparing the nucleation process in the Ni-5%W alloy substrates under different strain variables and the temperature decay caused when the cube texture reaches the same level is rarely reported. Therefore, we have carried out an in-depth study and analysis of the differences in the formation of differently oriented grains of Ni-5%W alloy substrate, which is initially recrystallized, and the texture evolution during the annealing process with a large strain using the electron backscattering diffraction (EBSD) technique. Elucidating the nucleation mechanism of recrystallization in metals undergoing intense rolling deformation will help us to further understand the relationship between recrystallization and rolling texture. It is hoped that these studies will provide better guidance on the quality of the alloy substrates used in coated conductors and establish a theoretical basis for their large-scale commercialization.

## 2. Materials and Methods

In the vacuum smelting process, the nickel block with 99.95% purity and the tungsten with 99.99% purity (according to the atomic ratio of 95:5) are re-cast at 1500 °C, and then the homogenization heat treatment is carried out at 1100 °C for 24 h to eliminate the internal stress. Finally, the initial ingot of Ni-5%at.W (abbreviated as Ni-5%W) alloy with thickness of 7 mm, length of 40 mm, and width of 30 mm is obtained. Before starting rolling, it is cut to the appropriate size using electric spark wire cutting technology, and the thickness remains unchanged. In the initial rolling stage, a two-high mill with a roll diameter of 150 mm is first used for rolling, with 5% deformation per pass. With the increase in deformation, the two-high mill reaches the working limit, and when the shape variable reaches 90%, the four-high mill with roll diameter of 50 mm is replaced for rolling. In the rolling process, we used combined stresses (von Mises strains, *ε*_vM_) to denote the rolling strain. The relationship between *ε*_vM_ and the form variable D can be expressed by the following equation:

$$\varepsilon_{vM}=\frac{2ln\left(1/\left(1-\frac{D}{100}\right)\right)}{\sqrt{3}}$$

where *D* is the amount of rolling deformation and $ln\left(1/1-\frac{D}{100}\right)$ represents the true strain during rolling. Therefore, the samples with strains of 3.9, 4.9, and 5.1 correspond to deformations of 96.7%, 98.5%, and 98.8%, respectively. The samples with different strains were heated at a rate of 5 °C/min. Subsequently, the samples were held at different temperatures for 1h for annealing, and a protective atmosphere of Ar-4% H2 was introduced to prevent oxidation, enabling a more accurate study of the texture evolution. Finally, the samples were cooled in the furnace.

The texture and microstructure of the Ni-5%at.W alloy substrate in the recovery and recrystallization stages were characterized using scanning electron microscopy (SEM, QUANTA FEG 450, EDAX, Troy, MI, USA) equipped with EBSD. The accelerating voltage for the EBSD scan was set to 20 kV, with a working distance of approximately 14 mm, and the spot size was set to 60 nm. The step size for the EBSD test was selected based on the grain size and test area. Consequently, the scanning step parameters were set to 0.12 μm for the recovery stage, 0.15 μm for the cross-section sample in the recrystallization stage, 0.7 μm for the surface sample, and 1.5 μm for the grain-growth stage.

Additionally, within the range of a 15° deviation angle, the EBSD orientation distribution map exhibited the same orientation structure. The grain orientation was analyzed in detail using TSL OIM Analysis 7 orientation analysis software. Low-angle grain boundaries (LAB) and high-angle grain boundaries (HAB) were defined as boundaries for orientation differences of 2–10° and >10°, respectively. The Σ3 boundary was defined by applying a maximum deviation of 3° from the ideal 60° orientation difference <111>.

The hardness of the Ni-5%at.W alloy substrate was measured using a Vickers hardness tester at different temperatures under a load of 200 g for 15 s, from which the recrystallization temperature of the substrate was determined. Additionally, X-ray diffraction (XRD, Bruker AXS D8 Advance, Cu target, Bruker Corporation, Carlsbad, CA, USA) was used to measure the texture of the samples, and Diffrac Plus.TexEval2.x software was employed to analyze the XRD data.

## 3. Results

**Figure 1:**
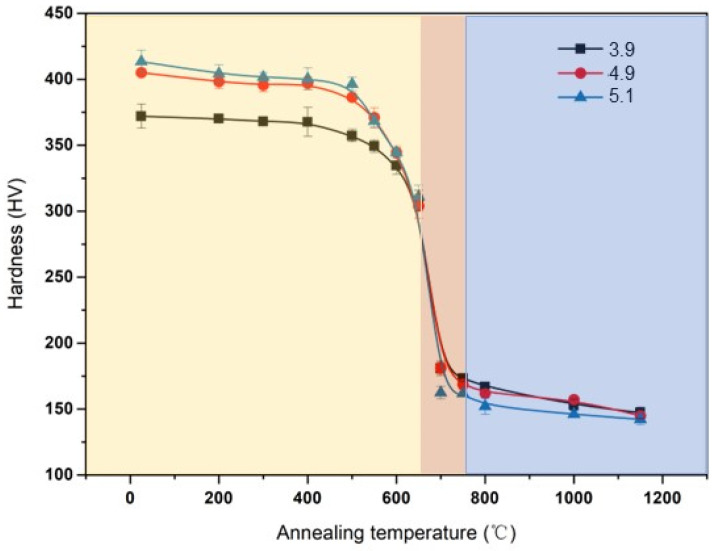
The hardness of the Ni-5%W alloy substrate with different strains.

The entire annealing process can be divided into three stages: recovery, recrystallization, and grain growth, based on the changes in the hardness curve of the Ni-5%at.W alloys during heat treatment. The stage of sharp decrease in hardness corresponds to the recrystallization stage. As shown in Figure 1, all curves undergo a sharp decline, and the hardness value drops to about 1/2 of the initial hardness at 650 °C. The decreasing rate of hardness value slowed down significantly after further heating, so 650 °C was determined as the initial recrystallization temperature point. This stage is preceded by a recovery stage and followed by a grain growth stage. Consequently, the evolution of microstructure and texture during the annealing of the Ni-5%at.W alloys with different strain levels has been thoroughly investigated.

### 3.1. Recovery Step

**Figure 2:**
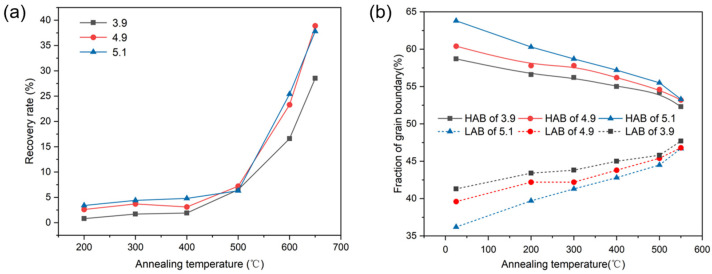
(**a**) Recovery rate of Ni-5%W alloy substrate at different strains; (**b**) Fraction of high and low grain boundaries in the recovery stage for Ni-5%W alloy substrate at different strains.

The recovery rate R during the recovery stage can be expressed as the reduction in hardness value at a given recovery temperature compared to the reduction in the total hardness value. The formula is as follows:

$$R=\frac{H_{0}-H}{H_{0}-H_{i}}\times 100\%$$

where *H*_0_ represents the hardness in the initial state, *H* denotes the hardness after heat treatment at 1150 °C, and *H*_i_ indicates the hardness at a specific temperature. As depicted in Figure 2a, the highest rate of return for the same heat treatment temperature is observed for strain 5.1, attributable to the higher deformation storage energy in the samples with higher strain.

**Figure 3:**
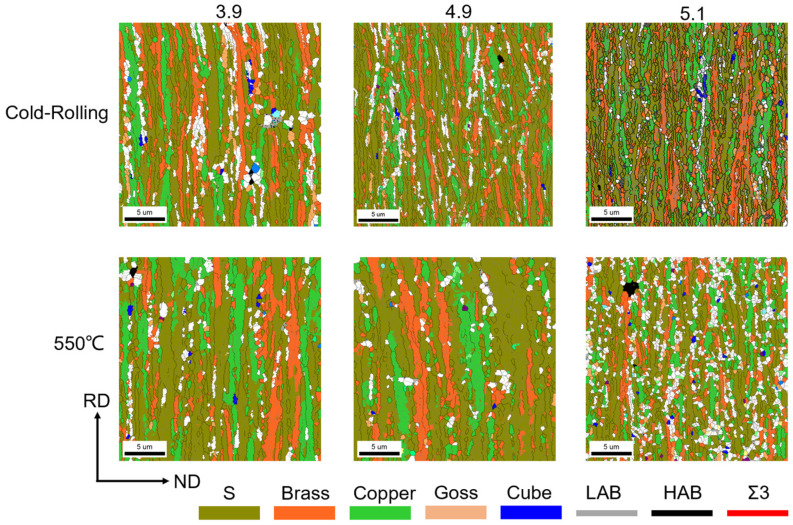
The grain orientation of Ni-5%W alloy substrate with different strains at 550 °C and cold rolling.

EBSD analyses were first conducted on the cross sections of the samples in the cold-rolled state and after 550 °C recovery (with strains of 3.9, 4.9, and 5.1). Upon observing the orientation map, it was noted that the microstructure of the Ni-5%W alloy substrate, after experiencing significant strain deformation, exhibited a lath-like morphology parallel to the rolling direction (RD), with the main dislocation substructures of the grains appearing as laminar dislocation interfaces (LBs), as depicted in Figure 3. It was observed that with increasing strain, the long lamellar structure along the RD direction became more refined, and the grain aspect ratio increased.

After annealing at 550 °C, the deformation resulting from cold rolling was partially recovered with increasing temperature. During this process, the long lamellar rolled tissue gradually coarsened and transformed into equiaxed tissue, and a portion of the long lamellar tissue disappeared during recovery. This suggests that some high-angle grain boundaries shifted during recovery, causing the length of the recovered tissue to decrease in the RD direction and increase in the ND direction, resulting in a significant decrease in the grain aspect ratio. In Figure 2b, the fraction of high- and low-angle grain boundaries of the Ni-5%W alloy substrate during the recovery process is plotted. A substantial number of high-angle grain boundaries exist in the initial cold-rolled state for all three samples. As temperature increases and the recovery stage extends, the fraction of high-angle grain boundaries decreases from 63.8% to 53.3% for the sample with strain 5.1, from 60% to 53% for the sample with strain 4.9, and from 58% to 52% for the sample with strain 3.9. The content of low-angle grain boundaries subsequently increases. The content of Σ3 twin boundaries represents a small proportion of the entire recovery phase, accounting for only about 5%, and does not change significantly as the recovery stage progresses. This suggests that the thermal stability of Σ3 twin grain boundaries is higher during the recovery process, while the thermal stability of high-angle and low-angle grain boundaries is lower, resulting in a higher migration rate. Some high-angle grain boundaries move during the heat preservation process, causing the grains to merge and disappear from each other, leading to a reduction in their contents.

**Figure 4:**
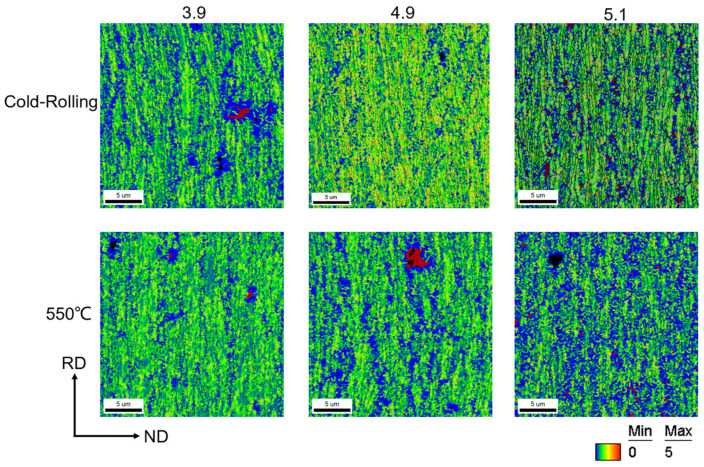
KAM maps of Ni-5%W alloy substrate with different strains at 550 °C and cold rolling.

In addition, the KAM (kernel average misorientation) map, measured by EBSD, is a model that represents the local microstrain by calculating the local deviation angle. Microstrain is closely related to the residual stress. The strain region is typically formed due to dislocation entanglement, and higher strain indicates a higher dislocation density, which in turn signifies higher residual stress. Therefore, the presence of residual stress in the sample can be observed through KAM. The KAM values of the initial cold-rolled state of all three samples are in the moderately high range and are uniformly distributed throughout the cross-section of the samples. The dislocations accumulated in the samples in the cold-rolled state after the 550 °C recovery were alleviated to some extent, as shown in Figure 4.

**Figure 5:**
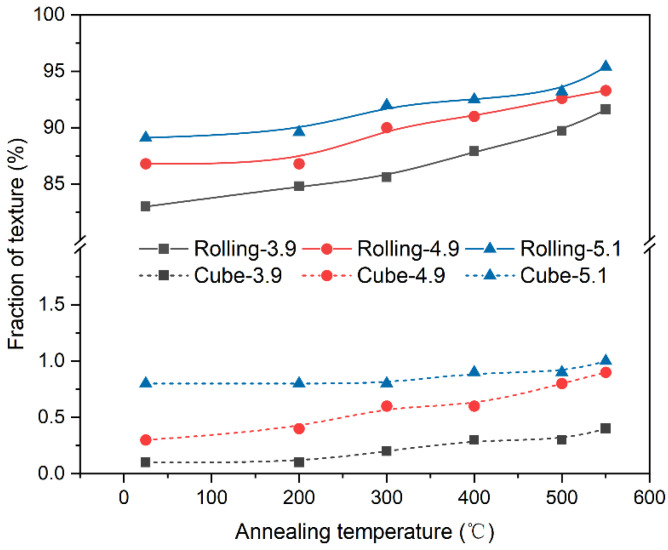
Rolling and cube textures of the Ni-5%W alloy substrate with different strains during the recovery step.

Figure 3 presents a grain orientation map depicting the evolution of the rolling texture (including S, Brass, and Copper) and the cube texture during the recovery stage. Although there is a slight enhancement of both the rolling and the cube texture during the recovery process, the rolling texture remains predominant. The texture data underwent statistical analysis, as shown in Figure 5. The results indicate that neither the rolling texture nor the cube texture changed significantly before 550 °C. The rolling texture was dominant in all samples, accounting for 91.6%, 93.3%, and 95.4%, respectively. Additionally, all three different strain samples exhibited less than 1% cube texture. Typically, after a certain level of deformation, over-rolling occurs. This means that the cube texture in the samples with high deformation does not have an overwhelming advantage but rather tends to decrease. In this experiment, the sample with a strain of 5.1 exhibited the relatively highest amount of cube texture, indicating that this critical value was not reached in this experiment. The EBSD plots after 550 °C recovery indicate that there were no obvious recrystallized grains in any of the three samples, and the grain size was significantly smaller in the 5.1 high-strain sample. In the EBSD orientation map, different colors represent different orientations: Cube{001}‹110›, S{123}‹634›, Brass{011}‹211›, Copper{112}‹111›, and Goss{011}‹100› orientations are represented by blue, grass green, orange, green, and light pink, respectively. Other random orientations not within these ranges are indicated by white.

### 3.2. Recrystallization Step

**Figure 6:**
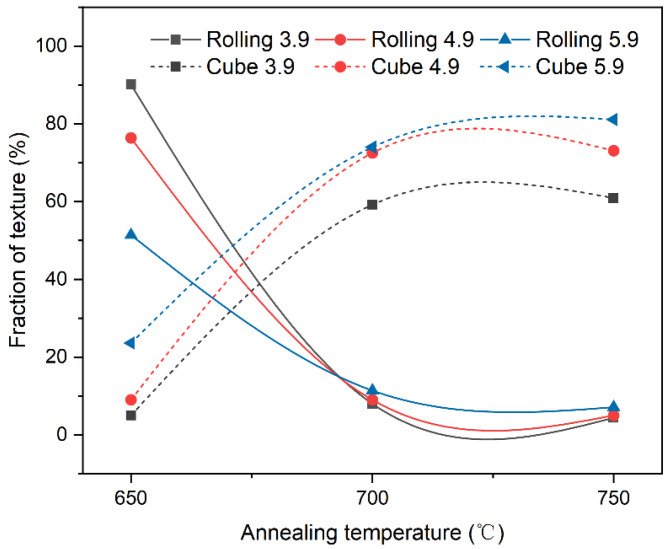
Rolling and cube textures of the Ni-5%W alloy substrate with different strains during the recrystallization step.

Recrystallization occurred in all samples when the temperature ranged from 650 °C to 750 °C. The recrystallization stage is a crucial factor to consider when exploring the formation and evolution of cube texture. As depicted in Figure 6, the texture fraction of the substrate with different strains varies significantly during the recrystallization stage. The fraction of rolling texture decreases rapidly in an exponential pattern, while the cube texture increases synchronously at an approximate rate. This indicates that the texture fraction of the substrates with three different strains follows the same evolutionary process during recrystallization.

**Figure 7:**
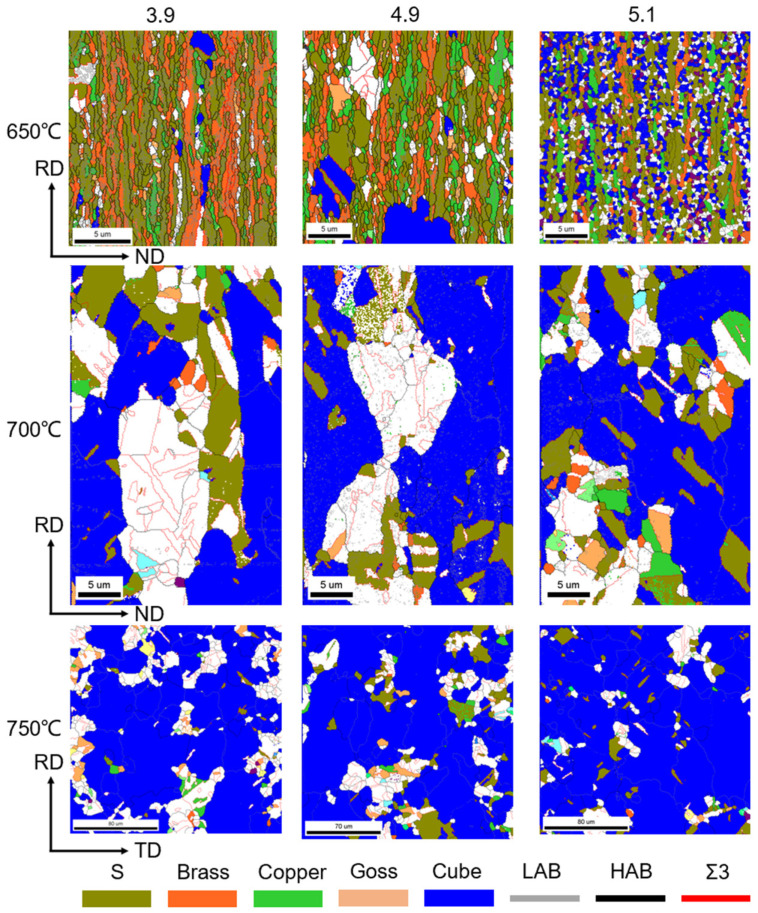
Grain orientation of Ni-5%W alloy substrate with different strains at 650–750 °C.

**Figure 8:**
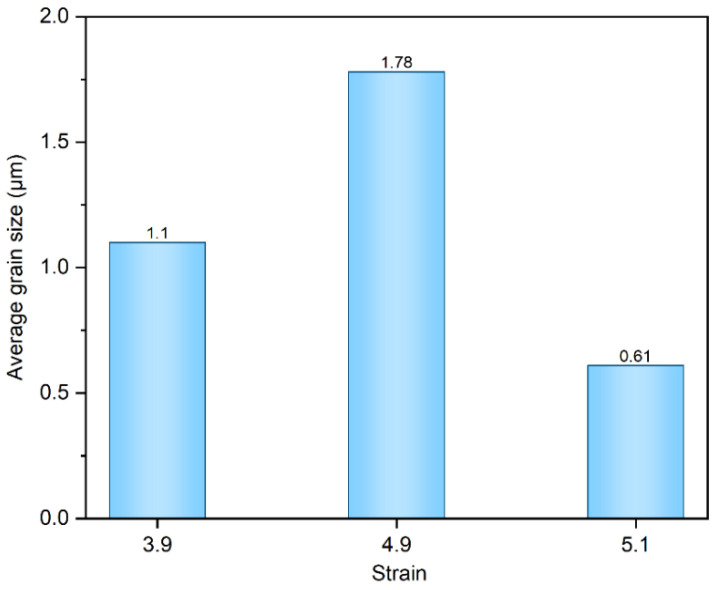
Average grain size of Ni-5%W alloy substrate with different strains at 650 °C.

Figure 7 depicts the orientation of the three strains at 650–750 °C. It is worth noting that there are significant differences in the recrystallization patterns of the three samples at 650 °C. This suggests that the recrystallization texture originates from the recrystallized grains of varying shapes. Both samples with strains of 3.9 and 4.9 exhibit recrystallization patterns that develop from long lamellar organizations, with the recrystallized grains having notably larger grain sizes along the RD direction. In contrast, the substrate with a strain of 5.1 develops its recrystallization texture from equiaxed crystals, resulting in a fragmented distribution. This implies that, in general, recrystallization in the Ni-5%W alloy substrate occurs directly in the long lamellar tissue. Additionally, when the strain reaches a certain level, the cube texture tends to emerge from the equiaxed crystals. At this temperature, the microstructures of the Ni-5%W alloy substrate samples with strains of 3.9, 4.9, and 5.1 contain recrystallized grains and rolling grains to varying degrees. As illustrated in Figure 8, the average grain sizes are 1.1 μm, 1.78 μm, and 0.61 μm, respectively, indicating that the grain growth phenomenon becomes increasingly significant with the increase of strain in the early stages of recrystallization. However, once a certain critical value is reached, the grain growth phenomenon diminishes.

After annealing at 700 °C, the samples were nearly fully recrystallized, and all three samples exhibited extensive recrystallization, rendering the earlier differences insignificant. The presence of rolling grains is minimal, with extensive twin boundaries and twins observable in all samples. The recrystallization process is completed as the annealing temperature reaches 750 °C. Cube grains begin to grow and incorporate other orientations rapidly. It is important to note that the area size of the EBSD test differs for the samples at 650 °C, 700 °C, and 750 °C. This variation occurs since all samples begin to recrystallize at 650 °C. At this temperature, most of the grains are still in the form of flakes of a few hundred nanometers, resulting in a small scanning area. At 700 °C, the recrystallized grains begin to grow and dominate, necessitating an enlarged scanning area to observe more regions. Although the scanning area size differs, both 650 °C and 700 °C represent RD-ND cross sections. At 750 °C, the RD-TD surface is measured because there are only three or four grains in the entire cross-section.

**Figure 9:**
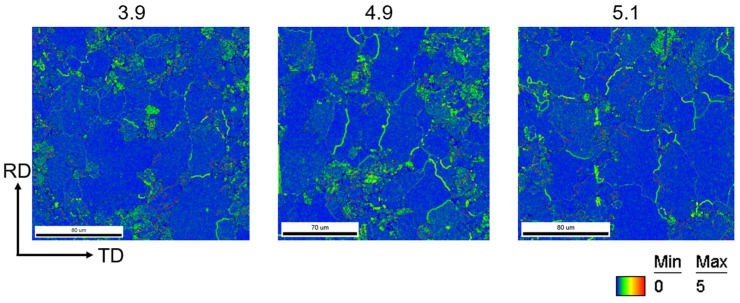
KAM maps of Ni-5%W alloy substrate with different strains at 750 °C.

After annealing all the samples at medium and high temperatures, their KAM values decreased significantly overall, with only medium to high KAM values remaining at grain boundaries, as depicted in Figure 9. This suggests that the recrystallization process has largely eliminated the residual stresses resulting from cold rolling in the samples.

### 3.3. Growth Step

**Figure 10:**
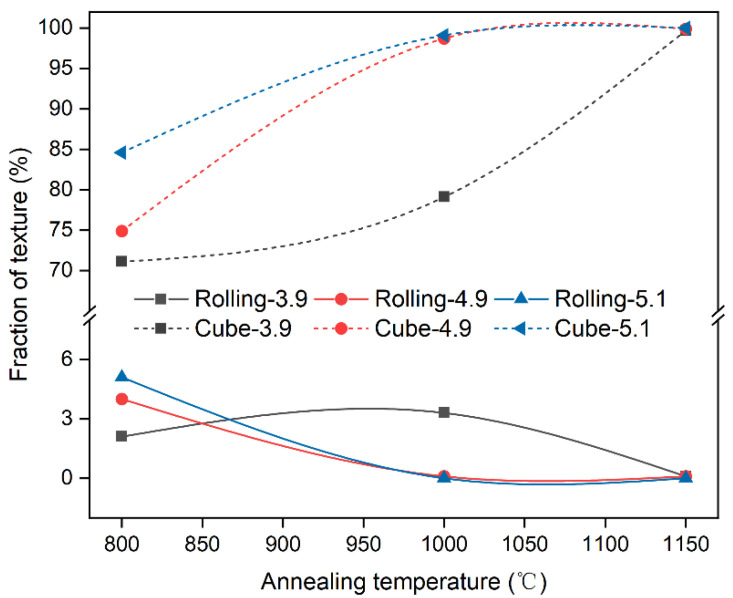
Rolling and cube textures of the Ni-5%W alloy substrate with different strains during the growth step.

In the annealing temperature range of 800 °C to 1150 °C, the samples with three different strain levels were in the growth stage. The preceding recrystallization process was accompanied by a large number of grain twins, involving not only the twinning of cube-oriented grains but also the twinning of rolling-oriented and other randomly oriented grains. Consequently, the recrystallized grains contain a significant proportion of randomly oriented grains. After the grain growth process, the cube texture remains dominant in all three samples, and the content of the cube texture continues to increase. Additionally, the higher the temperature, the lower the rolling texture content, as shown in Figure 10. Following annealing at 800 °C for 1 h, the Ni-5%W alloys with strains of 3.9, 4.9, and 5.1 exhibited 71.1%, 74.9%, and 84.6% of cube texture (<15°); 2.1%, 4%, and 5.1% of rolled texture (<15°); and 26.8%, 21.1%, and 10.3% of other random orientations, respectively.

In the later stages of grain growth, grains with cube texture grew and engulfed other oriented grains. The cube texture of the samples eventually reached approximately 99% for all three strains. Table 1 presents detailed microscopic information of the samples with different strains at 800 °C in the initial growth stage and 1150 °C in the late stage of grain growth. The average grain size and grain boundary content of the microstructure of all three samples were also roughly the same, with an average grain size of 50 μm after annealing at 1150 °C for 1 h. At this stage, the grain boundaries mainly consisted of low-angle grain boundaries, accounting for about 90% of the total, while the fraction of twinned grain boundaries decreased to less than 1%. Therefore, it can be concluded that high-temperature annealing has a limited impact on the final texture content and microstructure of Ni-5%W alloy substrate concerning strain. However, differences are observed in the evolution of texture and microstructure, which will be discussed in a subsequent section.

## 4. Discussion

Based on the above results, it is evident that we have systematically investigated the evolution of the microstructure and texture of the Ni-5%W alloy substrate with different strains throughout the heat treatment process. This contributes to a better understanding of the recrystallization mechanism of other face-centered cubic alloys. The experimental results demonstrate the formation of a strong cube texture (>99% content) in samples after undergoing a large strain rolling and high-temperature annealing process. Several points in this process require further discussion. Firstly, it is important to analyze whether the evolution of the cube texture occurs through the growth of cube nuclei formed during the recrystallization process or as a result of the annexation of the S-oriented and copper-oriented grains. Secondly, although the initial rolling texture contents are all high after large strain rolling and a strong cube texture is obtained in all cases after heat treatment, the cube texture generation curves of the samples with a strain of 5.1 tend to be in the high-temperature region throughout the evolution process, which is known as temperature decay.

**Figure 11:**
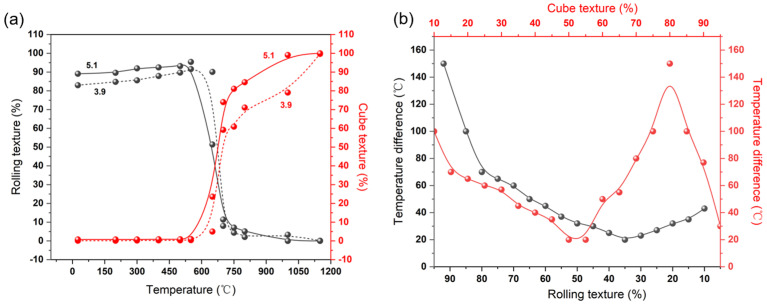
(**a**) Texture evolution results of Ni-5%W alloy substrate with different strain; (**b**) Temperature difference between the Ni-5%W alloy substrate with the strain of 3.9 and 5.1 when the texture fraction reached the same level.

Figure 11a depicts the curves illustrating the rolling texture and cube texture evolution of Ni-5%W samples with strains of 3.9 and 5.1 throughout the heat treatment process. The figure reveals that while there is a certain difference between the strains of the two samples, the trends of their evolution curves are very similar. However, the texture evolution curve of the 5.1 strain sample tends to skew towards higher temperatures compared to the 3.9 strain sample. It is hypothesized that this phenomenon is due to the disparity in the initial rolling texture and cube texture of each sample after cold rolling at different strains. The sample with a large strain of 5.1 exhibited a relatively high rolling texture after cold rolling, thus requiring a higher temperature to reduce the rolling texture content to the same level as the 3.9 strain sample. However, the primary factor influencing this temperature decay is the difference in the initial texture after cold rolling, which has minimal impact on the subsequent annealing process.

Figure 11b illustrates the temperature decay of the Ni-5%W samples with the same level of rolling texture and cube texture at strains of 3.9 and 5.1, respectively. The temperature decay trend of the rolling texture is relatively consistent overall, with only larger temperature differences of up to 150 °C observed at higher levels of rolling texture, primarily due to differences in the initial rolling texture. Additionally, the evolutionary dynamics of the rolling textures show minimal variation. However, the temperature decay between the cube textures of the two samples is notably different. Before the cube texture fraction reaches 50%, the temperature decay curves of the cube texture follow a similar trend to that of the rolling texture. Once the content exceeds 50%, the temperature decay tends to increase exponentially. However, after the cube texture fraction reaches 80%, the temperature decay rapidly decreases again. It is hypothesized that in addition to the effect of the initial texture after cold rolling, there are other factors influencing the evolution of the cube texture between samples with different strains. Based on these results, it is evident that the anomalies in temperature decay mainly originate in the nucleation stage, corresponding to 650 °C. It is imperative to conduct a more thorough analysis of the nucleation steps and texture evolution of these two samples at this temperature.

**Figure 12:**
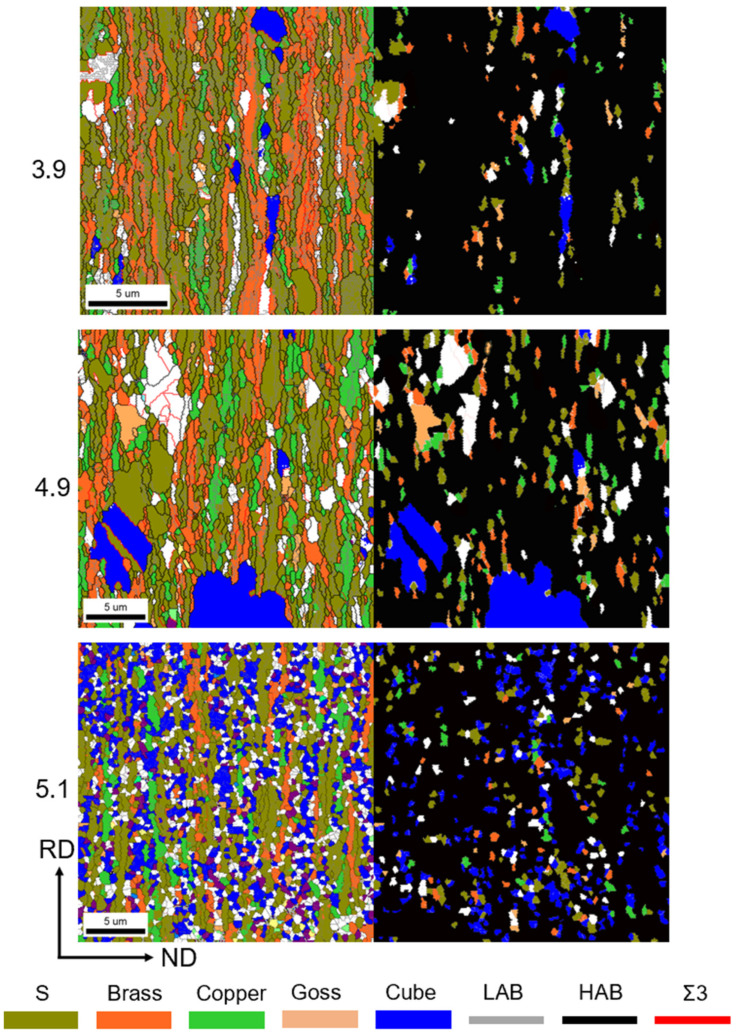
Grain orientation and nuclei map of Ni-5%W alloy substrate with different strains at 650 °C.

During the recrystallization nucleation process, the differences in the evolution of the 3.9 and 5.1 strain samples are significant, and it is hypothesized that the recrystallized nuclei produced by the different textures may be a crucial factor. Therefore, a simple division of textures into rolling textures and cube textures is no longer sufficient for a comprehensive and precise analysis of the influencing factors. In a subsequent study, we classified grain orientations as S, Brass, Copper, Goss, Cube, and random orientations. Before commencing the analysis, the recrystallized nuclei were defined [30] as follows: (1) nuclei with a size greater than 3 μm; (2) grains surrounded by high-angle grain boundaries greater than 1/3; and (3) intra-grain orientation difference < 0.1°. This classification is necessary because recrystallized grains must reach a certain size to overcome surface tension and be surrounded by large angular grain boundaries to exhibit high mobility. Figure 12 depicts the EBSD map and kernel map at 650 °C for different strains. The orientation diagram is shown on the left side of Figure 12. The nuclei map is shown on the right side of Figure 12, where only the recrystallized nuclei with different orientations are highlighted in color, while the rest are shown in black, enabling clearer observation of the recrystallized nuclei. The results indicate that all three strain samples exhibit recrystallized nuclei with different orientations, but their shape, size, and distribution are significantly different. These differences may be the primary reason for the substantial increase in the degree of temperature decay. Firstly, the location of recrystallized nuclei formation varies. In the 3.9 and 4.9 strain samples, the recrystallized nuclei originated from the lamellar tissue, with predominantly striated grain shapes and clear dominance in individual grain sizes. In contrast, the 5.1 strain samples exhibit significantly different nucleation locations, with recrystallized nuclei growing from equiaxed crystals and no apparent difference in grain size. Additionally, the nuclei are uniformly distributed rather than concentrated in one place. This phenomenon is attributed not only to the difference in initial strain but also to variations in their recovery organization.

**Figure 13:**
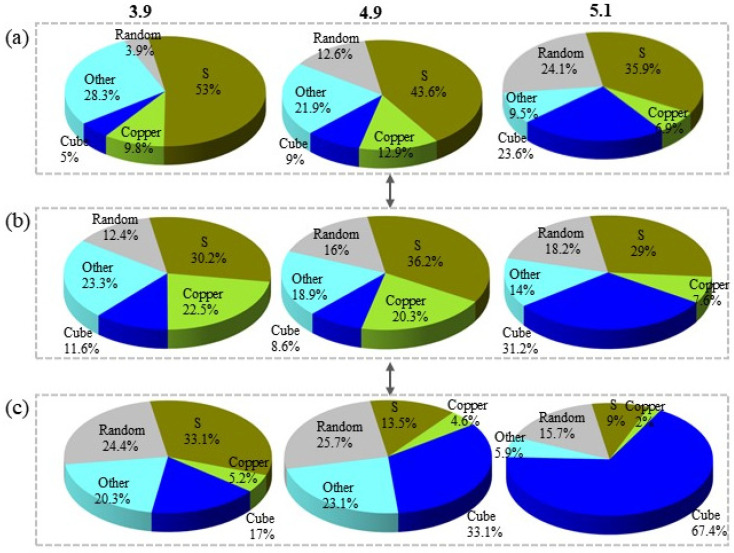
EBSD data of Ni-5%W alloy substrate with different strains at 650 °C: (**a**) the texture fraction, (**b**) nucleation grain, (**c**) grain area.

**Figure 14:**
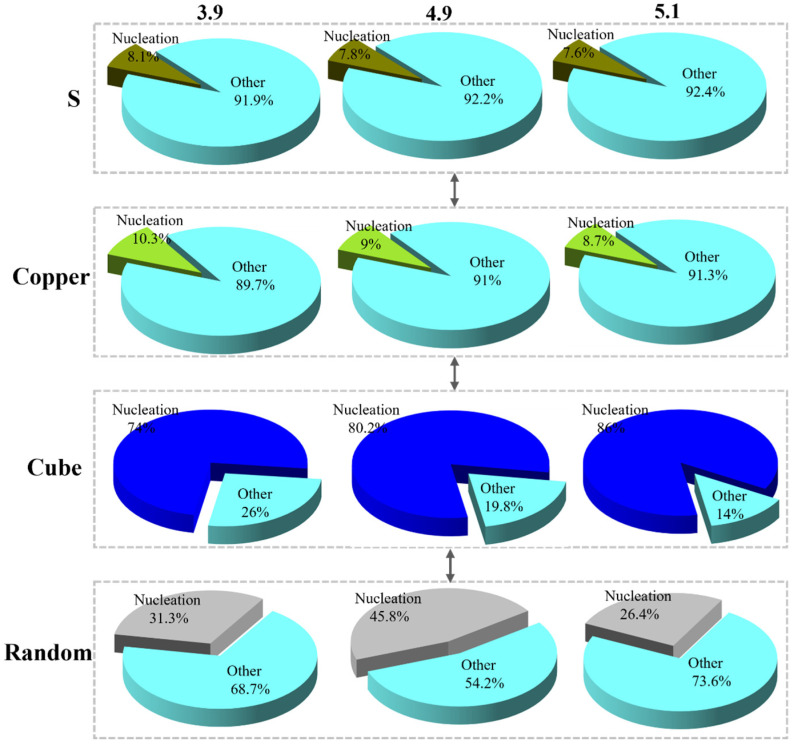
The ratio of nucleation in the whole texture at 650 °C.

In addition to the nucleation location of the recrystallized nuclei, the fraction of each orientation texture in the recrystallized grains is also worth considering. Figure 13 presents the fraction of grain content, the number of nuclei, and the area of nuclei in each orientation as measured by EBSD. In this case, the Goss orientation is combined with the Brass orientation as “Other” due to its lower fraction. At 650 °C, the cube texture fraction of the sample with a strain of 3.9 was 5%. As the strain increases, the fraction of cube texture also increases, with the fraction reaching 23.6% for the sample with strain 5.1. The number of nuclei in each orientation of recrystallization is not significantly different from the overall texture, with the S-orientation occupying a large fraction. Regarding the statistics of the area of the recrystallized nuclei of each orientation, the fraction of S-orientation decreases with increasing strain, while the fraction of cube-orientation nuclei area and random-orientation nuclei area increases. The increase in the fraction of randomly oriented texture is attributed to the generation of a large number of twinned grains, including randomly oriented grains, during the recrystallization process. Consequently, the area of randomly oriented grain nucleation occupies 24.4%, 25.7%, and 15.7% of the samples with strains of 3.9, 4.9, and 5.1, respectively. Figure 14 depicts the fraction of recrystallized grains of each orientation to the grains of each orientation. According to this statistic, the fraction of recrystallized grains in S-orientation and Copper-orientation is below 10% for different strains. In contrast, the fraction of recrystallized grains in the cube texture is above 70%, and the fraction of recrystallized grains in the random orientation texture is also around 30%. This indicates that at 650 °C, the vast majority of the cube and random orientation grains begin to recrystallize, and the cube-oriented recrystallized grains increase with increasing strain.

The number of nuclei (*N*_A_), nucleation rate (*N*_V_), and average grain size (*r*) of the recrystallized grains for each orientation are tabulated in Table 2. The nucleation rate was calculated as follows [30]:

$$\frac{N_{A}}{\gamma }\times k=N_{V}$$

Here, *K* represents a geometric constant. The samples with strains 3.9 and 4.9 exhibit 4.9 and 12 times more cube nuclei, respectively, compared to the sample with strain 5.1. However, the number of cube nuclei in the sample with strain 5.1 is 3.5 and 2.6 times larger than that of the samples with strains 3.9 and 4.9, respectively. Additionally, as indicated by the nucleation rate, most of the grains in sample 5.1 are in a nucleated state. In contrast, the cube grains in samples 3.9 and 4.9 have begun to grow.

**Figure 15:**
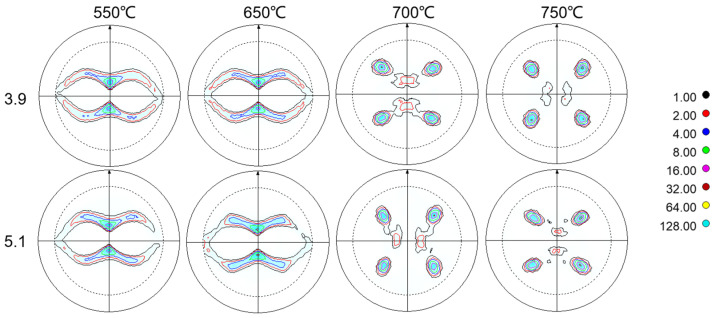
(111) Polar plots of Ni-5%W alloy substrate at the nucleation stage with different strains.

In the above analyses, we observe that the Ni-5%W alloy substrate samples with different strains exhibit some degree of variation. The evolution of the texture of the Ni-5%W alloy in the late stage of recovery and recrystallization was tested and analyzed from a macroscopic point of view using XRD four-ring diffraction. As depicted in the (111) pole figure in Figure 15, the overall evolutionary trend of the macroscopic texture of samples 3.9 and 5.1 is not significantly different. Following the recovery stage, all samples exhibit a classic copper-type texture internally. The texture then begins to transform at 650–700 °C, leading to a transition-type texture. Ultimately, at the end of the recrystallization stage, all samples have completed the transition from a rolling texture to a cube texture.

**Figure 16:**
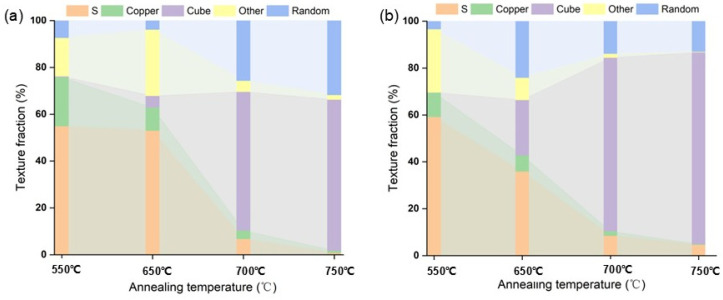
Texture evolution in the nucleation step of the Ni-5%W alloy substrate with different strains. (**a**) 3.9, (**b**) 5.1.

Figure 16 provides a detailed illustration of the evolution of textures throughout the growth stage, from the late stage of recovery at 550 °C to recrystallization. The nucleation of cube grains in both samples after annealing at 550 °C is below 1%. Recrystallized grains begin to nucleate after annealing at 650 °C for 1 h. Some of the recrystallized grains in samples 3.9 and 4.9 have already entered the growth stages, as indicated by the EBSD maps in Figure 12. Samples with strain 5.1 begin to nucleate extensively. Still, all the nucleation sites of the 5.1 samples originate from the equiaxed crystals compared to the samples with lower strain. In summary, it can be inferred that the number of nucleations and whether the cube grains have grown or not are among the main factors affecting the temperature decay produced by the cube texture between different strains. Additionally, Figure 16 suggests that the fraction of random texture appears to increase during the recrystallization process due to the growth of many annealed twins. Cube textures and randomly oriented textures are closely related in this process, and both grow by engulfing the rolling textures, mainly S textures and Copper textures. The sample with a strain of 3.9 exhibits a leading step in the growth of cube grains at 650 °C, suppressing the increase in randomly oriented textures. Subsequently, the cube texture and the randomly oriented texture compete with each other to engulf the rolling grains. On the other hand, samples with a strain of 5.1 are all in the nucleation stage of cube grains at 650 °C and do not have a significant size advantage. However, when the temperature reaches 700 °C, the cube-oriented grains begin to engulf the randomly oriented grains, leading to a decrease in the fraction of randomly oriented texture from 24.1% to 13.9%.

In conclusion, the main factors influencing the temperature decay of the cube texture in samples of different strains have been identified. In samples with lower strain, the nucleation of cube grains occurs in larger-sized lamellar tissue, providing a significant size advantage and inhibiting the increase of randomly oriented grains during nucleation. Conversely, in the high-strain samples (5.1), the cube texture nuclei originate in equiaxed crystals with radial direction (RD) sizes similar to normal direction (ND) sizes. These crystals are fragmented and uniformly distributed, but their smaller size results in no size advantage. The cube texture grows by competing with the randomly oriented texture, which, in turn, engulfs the randomly oriented grains. These differences have a significant impact on the temperature decay of the cube texture across different strains.

## 5. Conclusions

In this study, we systematically investigated the nucleation process of the Ni-5%W alloy substrate and the evolution of cold rolling texture in the stages of recovery (25–550 °C), recrystallization (650–750 °C), and grain growth (800–1150 °C) at various large strain levels. The 3.9 and 4.9 strain samples exhibited a more concentrated distribution of cube texture growth in the lamellar tissue. Additionally, the cube grains tended to grow earlier compared to the 5.1 strain sample. In contrast, the cube texture in the 5.1 strain sample originated in equiaxed crystals with little difference in size in the radial direction (RD) and normal direction (ND) and was uniformly distributed in a fragmented manner. When the grain size of the 5.1 strain sample was 650 °C, the cubic grains were all in the nucleation stage, and when the temperature reached 700 °C, the cubic-oriented grains began to grow and gradually absorb the randomly oriented grains. After annealing at 1150 °C for 1 h, a strong cube texture was achieved in all strain samples, with the texture content exceeding 99%. However, texture evolution during the annealing process differed among the various strain samples. Furthermore, the emergence of annealing twins led to an increase in the content of randomly oriented textures, which competed with cube textures to engulf rolling textures. Compared to the 3.9 strain sample, the 5.1 strain sample exhibits a higher nucleation rate and faster growth rate of cubic grains, and the differences in strain result in an abnormal increase in temperature variation when the cubic texture content of the Ni5W alloy base is the same during the nucleation stage.

## Figures and Tables

**Table 1:** Detailed information on the texture, grain boundary, and average grain size of the Ni-5%W alloy substrate with different strains at 800 °C and 1150 °C.

Scheme	Temperature (°C)	Average Grain Size (μm)	Grain Orientation (%)			Grain Boundary (%)		
			Rolling	Cube	Random	LAB	HAB	Σ3
3.9	800	7.3 ± 7.8	2.1	71.1	26.8	13.7	61.9	24.4
	1150	50.0 ± 84.1	0.1	99.7	0.2	89.4	10.2	0.4
4.9	800	7.5 ± 6.7	4	74.9	21.1	23.2	47.1	29.7
	1150	55.7 ± 89.9	0.1	99.9	0	91.3	8.7	0.6
5.1	800	7.6 ± 7.3	5.1	84.6	10.3	28	50.5	21.5
	1150	54.9 ± 76.0	0	100	0	90.3	9.5	0.2

**Table 2:** Number of the recrystallized grains, nucleation ratio, and average grain size of different orientations.

Strain		S	Brass	Copper	Goss	Cube	Random
3.9	N_A_	39	20	29	10	15	16
	r	0.59	0.56	0.49	0.46	2.68	0.62
	N_V_	19.17	10.36	17.16	6.3	1.62	7.48
4.9	N_A_	84	35	47	9	20	37
	r	0.74	0.68	0.71	0.79	6.76	3
	N_V_	32.92	14.93	19.20	3.3	0.86	3.58
5.1	N_A_	91	29	24	15	98	57
	r	0.9	0.53	0.59	0.52	0.55	0.54

## Data Availability

The original contributions presented in the study are included in the article, further inquiries can be directed to the corresponding authors.
